# What we know and don’t know about the immunization program of Ethiopia: a scoping review of the literature

**DOI:** 10.1186/s12889-020-09304-1

**Published:** 2020-09-07

**Authors:** Binyam Tilahun, Zeleke Mekonnen, Alyssa Sharkey, Asm Shahabuddin, Marta Feletto, Meseret Zelalem, Kabir Sheikh

**Affiliations:** 1 Department of Health Informatics, Institute of Public Health, College of Medicine and Health Sciences, University of Gondar, Gondar, Ethiopia; 2 eHealthLab Ethiopia, Institute of Public Health, College of Medicine and Health Sciences, University of Gondar, Gondar, Ethiopia; 3 Health System Directorate, Ministry of Health, Addis Ababa, Ethiopia; 4 Implementation Research and Delivery Science Unit, UNICEF Health Section, New York, USA; 5 Alliance for Health Policy and Systems Research, World Health Organization, Geneva, Switzerland; 6 Maternal and Child Health Directorate, Ministry of Health, Addis Ababa, Ethiopia

**Keywords:** Immunization, Vaccination, Immunization program, Review, Ethiopia

## Abstract

**Background:**

There has been significant recent prioritization and investment in the immunization program in Ethiopia. However, coverage rates have stagnated and remained low for many years, suggesting the presence of systemic barriers to implementation. Hence, there is a need to consolidate the existing knowledge, in order to address them and consequently improve program effectiveness.

**Methods:**

A thorough literature review and Delphi method were used. In this review, we searched Pubmed/Medline, WHO library, Science direct, Cochrane library, Google scholar and Google using different combinations of search strategies. Studies that applied any study design, data collection and analysis methods related to immunization program were included. In the Delphi method, a panel of 28 national and international experts were participated to identify current evidence gaps and set research priorities under the immunization program.

**Results:**

In this review, a total of 55 studies and national documents were included. The review showed that the vaccination coverage ranged from 20.6% in Afar to 91.7% in Amhara region with large inequities related to socio-economic, health service access and knowledge about vaccination across different settings. Only one study reported evidence on timeliness of immunization as 60%. The review revealed that 80% of health facilities provide immunization service nationally while service availability was only 2% in private health facilities. This review indicated that poor vaccine storage, vaccine shortage, service interruptions, poor defaulter tracing, low community engagement and poor documentation were the main barriers for the Expanded Program on Immunization with variations across different regions. Through expert panel of discussion using Delphi method, 10 priority research areas were identified across different domains of the immunization program at national level.

**Conclusion:**

We found out that there is substantial knowledge on vaccination coverage, however, there is little evidence on timeliness of vaccination. The existing barriers that affect full immunization coverage also varied from context to context which indicates there is a need to design and implement evidence based locally tailored interventions. This review also indicated evidence gaps with more focus on health system related implementation barriers at lower level and identified further research priorities in the immunization program of Ethiopia.

## Background

Immunization is one of the main health interventions to prevent childhood morbidity and mortality [1, 2]. The Expanded Program on Immunization (EPI) in Ethiopia, launched in 1980, has been one of the core priorities in the current Health Sector Transformation Plan (HSTP). The health development army (HDA) plays a critical role in mobilizing communities for immunization and identifying children who do not return to complete their vaccinations [3, 4].

Vaccines are provided routinely in health facilities all over the country in static, out-reach and mobile health facilities. In addition, campaigns are in place since 2011 providing polio, measles and other antigens to children through improved district planning and with a goal of reaching every district (RED). The EPI program currently provides 11 antigens targeting major childhood killer diseases during the child’s first year [4].

Immunization becomes more effective if a child receives the full course of recommended immunization doses. Though there has been a tremendous effort in Ethiopia, immunization coverage rates stagnated and remained very low for many years as stated in the Ethiopian Demographic and Health Survey (EDHS) report with full vaccination coverage of 39% in 2016. In spite of the promising progress, much more is required to achieve maximum optimization, effectiveness and protection [2]. To effectively control vaccine preventable diseases (VPDs), high immunization coverage is required with the target of the WHO to reach 90% coverage. In addition, age appropriate vaccination is also necessary for the success of the EPI program in Ethiopia [4].

Maintaining high performance and quality with in an immunization program is challenging. As a result, substantial proportions of children in many countries still fail to benefit from all basic vaccines and VPDs still pose a public health risk with the highest rates of child mortality still in Sub-Saharan Africa including Ethiopia [4]. Overall vaccine coverage is typically used as a metric to evaluate the adequacy of vaccine program performance, though it does not account for untimely administration, which may unnecessarily prolong children’s susceptibility to disease [5].

In Ethiopia, the RED strategy has been implemented in selected districts with poor vaccination coverage since 2004. To achieve the 2020 targets and deliver effective immunization services to every child, Ethiopia has developed different policies, strategies and plans including HSTP, comprehensive multiyear immunization plans (cMYP) and other supporting strategies The cMYP encompasses all components of immunization services: service delivery, vaccine supply, quality and logistics, disease surveillance and accelerated disease control, advocacy, social mobilization and communication and program management [4].

The strategies and strategic plans are translated into action through operational plans [4]. However, translating the strategies and plans into action is not easy due to different challenges including access, utilization, service delivery approaches, demand for immunization, community engagement and quality of services are key factors preventing immunization service delivery to reach every child. There is also limited understanding about immunization system barriers, facilitators and insufficient information on implementation bottlenecks which hinder effective immunization coverage [4, 6, 7]. In response to this problem, a review of available published and grey literatures was conducted.

## Rationale of the review

Though vaccination coverage in Ethiopia has increased steadily over time, it is not at the pace required to reach national and international targets. Moreover, the EPI program is challenged with large disparities in vaccination rates across geographic areas and population groups [2, 4]. Considering the situation, little is known about the implementation challenges and their underlying causes of EPI program in Ethiopia. In order to fill this gap, this scoping review of literature contributed to understand the current state of knowledge on the implementation of the EPI program. This scoping review also identified important implementation gaps and prioritized future research areas of immunization program in Ethiopia.

## Objectives

### General objective

This scoping review of literature aimed at exploring the current state of knowledge on the health system barriers affecting implementation of routine immunization program in Ethiopia. In addition, the review indicated the possible health systems research areas which need critical insight and further investigation in Ethiopian context.

### Specific objectives

The specific objectives of the review are:

- To explore the current state of knowledge on the implementation of the national immunization program
- To identify the barriers affecting implementation of immunization program
- To identify current knowledge gaps and prioritize potential research areas in the immunization program of Ethiopia

## Methods

### Literature searching and searching methods

We searched electronic databases like MEDLINE/Pubmed, WHO Library, Science Direct, Cochrane /Wiley Library, Google Scholar and Google. The review included studies/reports published from 1993 to 2018. The searching of literatures has been completed on November 28, 2018.

We used different combinations of keywords and texts to build the search strategy and identify relevant articles. The searching techniques considered Boolean operators with the following search terms.

“(Immunization OR vaccination OR Expanded program of immunization) AND (Facilitators OR Enablers OR challenges OR Barriers) AND (Infants OR Pediatrics OR Child OR Preschool) AND Ethiopia”.

In addition; unpublished papers, manuals, guidelines and reports from Ethiopian Federal Ministry of Health (FMOH) were searched and included for this review.

### Studies selection criteria

**Inclusion criteria**

- Studies on routine child immunization in a community or healthcare setting in Ethiopia
- Studies that applied any study design, data collection and analysis methods related to EPI
- Both published and unpublished studies that focused on implementation of EPI
- Administrative reports and national estimates which highlighted gaps or implementation challenges of EPI in Ethiopia
- Studies or reports with accessible full text

### Data extraction and management

Data were extracted using a standardized data extraction spread sheet. The data extraction sheet included study characteristics such as: authors’ name, publication year, study design, study setting, study population, proportion of vaccination coverage and timeliness. Data extraction was done by the two authors (Binyam Tilahun and Zeleke Mekonnen) independently. The level of agreement between the two reviewers were measured using Cohen’s Kappa level of agreement. The two authors’ resolved disagreements by discussion consulting a third author (Meseret Zelalem) for any persistent disagreements.

### Data analysis

The analytical discourse focused on reviewing and summarizing immunization coverage, dropout rates, service availability, cold chain system and other health system barriers for immunization coverage which deserve further concerted attention.

### Expert panel to identify research priorities

To identify research priorities under the immunization program, Delphi method was used. Accordingly, a group of 28 experts affiliated with different organizations (WHO, UNICEF, GAVI, MOH, RHBS, Universities, Health facilities) were involved in identifying and prioritizing the research questions for the immunization program in Ethiopia.

## Results and discussion

**Fig. 1:**
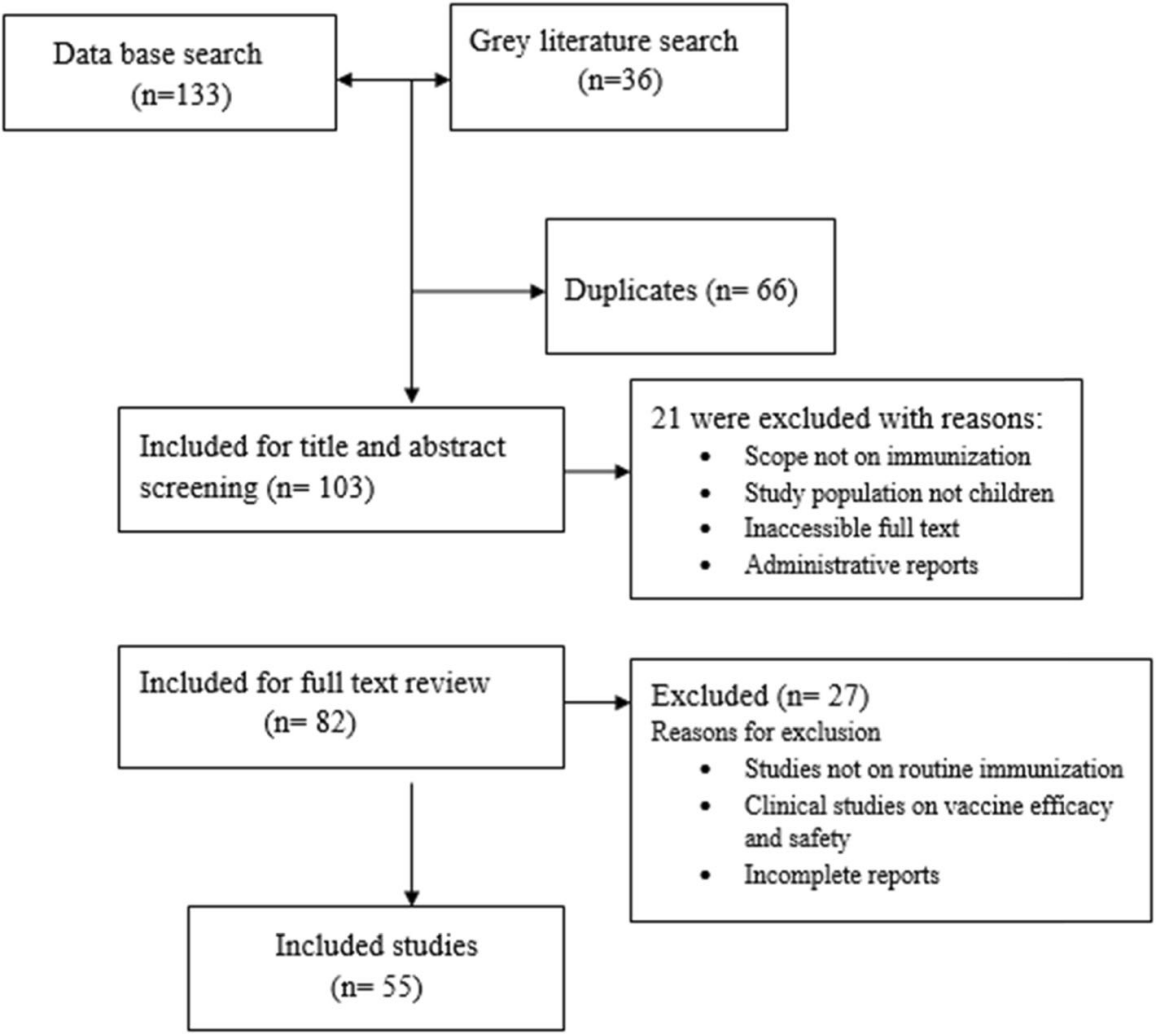
Study selection process

We included 55 studies and national documents related to immunization programs in Ethiopia (Fig. 1). Most of the studies were published and cross sectional by study design. Of those included studies, 4 were EDHS and 2 were National EPI coverage surveys. The remaining studies were conducted in different regions of the country since 1993. In addition, unpublished administrative Health Management Information System (HMIS) reports and national documents were included for the review.

Results were summarized along the following themes: Immunization coverage and timeliness, determinants of immunization service utilization, health service availability, supply chain management, EPI information systems, community engagement and gender inequalities.

### Evidence on utilization of immunization services in Ethiopia

#### Immunization coverage and timeliness

At national level there were six national level surveys conducted to assess immunization coverage; four EDHS studies [8–11] and two National EPI coverage surveys [5, 12]. In addition three FMOH administrative reports [13–15] and two WHO/UNICEF reports were reviewed [16, 17]. These studies showed an upward trend in immunization coverage in recent years in Ethiopia. The recent EDHS 2016 report indicated that the national immunization coverage has reached 39% from the coverage reported in 2000 (14%) [11]. However, regional disparities exist since 2000 till now where emerging regions have very low immunization coverage consistently. There are also marked urban-rural differences in vaccination coverage over time. The full immunization coverage rates included in the EDHS surveys were found to be far below EPI coverage survey findings, administrative reports and WHO/UNICEF estimates [13, 14, 16, 17] [Table 1]. From the review findings, the overall access to vaccination services was low. Access to vaccination was lowest in the Afar and Somali regions [5, 10, 11]. The dropouts from immunization were not in the acceptable range evidenced by the recent EDHS report (20% for Penta). This dropout rate is very high as compared to the target set for 2020 under the comprehensive multiyear plan [4] [Table 1]. The percentage of children who have received no vaccination was also consistently high and stagnant from 2000 (17%) to 2016 (16%) [8, 11].

**Table 1:** National Evidence on Full immunization coverage and timeliness in Ethiopia

S.N	Author	Design	Sample	Topic	Major findings	Conclusions
1	CSA, USAID (2000)	Cross sectional	2143	National EPI coverage survey report in Ethiopia	• DPT I 40% and DPT III 18% • 14% full (0% in Afar and 74% in AA) o Urban 42% and Rural 11% • 17% Not vaccinated	• Substantial differences in the coverage between regions • High dropouts
2	CSA, USAID (2005)	Cross sectional	1, 877	National EPI coverage survey report in Ethiopia	• DPT I 58% and DPT III 32%, • 20% fully (Afar 0.6% and AA 70%) • 24% No vaccination	• High dropout rates • Many unvaccinated children
3	Kidane T(2006)	Cross sectional survey	6903 children	National EPI coverage survey report in Ethiopia	• DPT I 84.3% and DPT III 66% • Fully 49.9% (Somali 14% and AA 87%) • Timely coverage of 20%	• Progress was not uniform in all regions of the country • Dropout rate was high
4	CSA, 2011	Cross sectional survey	1927	National EPI coverage survey report in Ethiopia	• 24% fully vaccinated (Afar 8% and AA 78%) o Urban 48% and rural 20% • 16% No vaccinations	• Disparity between regions • High dropout rate
5	EPHI (2012)	Cross sectional survey	3762	National immunization coverage survey	• Receiving all basic vaccination is 50% o Afar and Somali 12.6% while AA 94% • Valid dose of 18.6%	• Access and utilization is low in most regions • High drop-out rates
6	FMOH (2014)	HMIS	National	Policy and practice information for action	• Full 77.7% and Penta II 87.6%	• Relatively good coverage
7	CSA, USAID (2016)	Cross sectional	2004 children	Ethiopian demographic and health survey	• 39% fully (Afar 15% and AA 89%) • 22% were vaccinated timely • No vaccinations 16%	• The EDHS surveys have shown a steady progress in EPI coverage
8	FMOH (2015)	HMIS	National	Health and health related Indicators: 2016	• Penta III 94.4% and Fully 86.6%	• Showed good progress since 2010 coverage of 86%
9	WHO/UNICEF (2017)	Estimate	National	WHO and UNICEF estimates of immunization coverage: 2017 revision	• DPT I 85% and DPT III 73% in 2017	• Showed progress from previous estimates
10	FMOH (2018)	HMIS	National	Annual Health Sector Performance report	• Penta III 96% and full coverage 87% • Pent1 to measles drop-out was 13%	• Showed progress

There were also 15 pocket studies that determined immunization coverage in different regions of the country. Among them two were done in Oromia region with full vaccination coverage of 22.9% [18] and 36% [19], while five were done in Amhara region with full immunization coverage ranging between 58.4 and 91.7% [20–24]. Single study done in Afar [25], Somali [26] and Tigray [27] regions showed that the full vaccination coverage was 20.6, 36.6 and 51% respectively. The remaining four studies were conducted in Southern Nations and Nationalities (SNNP) region which showed immunization coverage ranging from 18.4 to 73.2% [28–31]. Studies were not found from Gambella and Benshangul- Gumuz regions. A survey by USAID in four regions of the country also revealed that full immunization coverage was better than the findings of majority of the studies (69%) [6]. The studies generally showed that the vaccination coverage in majority of the studies were low and the progress was not uniform across different regions of the country. Differences in coverage could be attributed by differences in the sampling frame, design, sample size, representativeness of the sample, and selection methodology, as well as differences in the source of information. Similarly, the Penta3 coverage was much lower than the Penta I coverage in all the studies with unacceptable range of dropout rates resulting in higher number of partially vaccinated children. The percentage of children who have received no vaccination also varied from study area to study area much worsening in SNNP region [28, 29]. The results reported from these surveys were generally lower than the administrative reports and national estimates [14, 16] [Table 2].

**Table 2:** Local evidence on immunization coverage and timeliness of immunization in Ethiopia

S.N	Author	Design	Sample	Topic	Study area	Major findings	Conclusions
1	Kidane T (2000)	Cross sectional	220	Factors influencing child immunization coverage in a rural District of Ethiopia	Tselemti district, Tigray Ethiopia	• 51% full coverage • BCG to measles defaulter 23.9%	High dropout rate
2	Beyene E (2006)	Cross-sectional	740	Factors associated with immunization coverage	Zone 3 of Afar Regional State	• Full immunization coverage was 20.6%	Low immunization coverage
3	Hussien M (2010)	Cross sectional	168	Assessment of Child Immunization Coverage and Associated Factors in Oromia Regional State, Eastern Ethiopia	Kombolcha district, Oromia	• 24.2% not immunized, • 52.9% partial and 22.9% fully • PentaI 73.8% % Penta III 33.1%	Low coverage High dropout rate
4	Belachew E (2011)	Cross sectional	536	Factors associated with complete immunization coverage	Ambo Woreda, Central Ethiopia	• 36% fully vaccinated • 23.7% unvaccinated	Low coverage
5	Waju B(2012)	Cross sectional	655 children	Childhood immunization coverage in Tehulederie district	Tehulederie district	• 83.1% of children were fully • 14.7% partially vaccinated	Relatively high coverage
6	Ayal D (2013)	Cross sectional	497	Assessment of fully vaccination coverage and associated factors in Mecha district	Mecha district, North West Ethiopia	• 49.3% were fully immunized • 1.6% c were not vaccinated	Coverage remains very low in the district
7	Amanuel D (2013)	Cross sectional	981	Determinants of Full Child Immunization; Evidence from Ethiopia	SNNP	• 81.6% children were not fully vaccinated	Low coverage
8	Abdi N (2014)	Cross sectional	582	Assessment of Child Immunization Coverage and Associated Factors in Oromia Regional State, Eastern Ethiopia	Jigjiga District, Somali Regional State, Ethiopia	• 74.6% were ever vaccinated • 36.6% were fully vaccinated	Coverage was found to be low
9	Mastewal W(2014)	Cross sectional	724	Factors for Low Routine Immunization Performance Dessie Town, Ethiopia	Dessie Town, Amhara, Ethiopia	• Full coverage 65.2% • 17.9% never get vaccine	Low coverage
10	Worku A (2014)	Cross sectional	630	Expanded program of immunization coverage and associated factors	Arba Minch town and Zuria District	• 73.2% fully, 20.3% partially and 6.5% received no vaccine	Better than the national immunization coverage
11	Melkamu B (2015)	Cross-sectional	751	Level of immunization coverage and associated factors among children	Lay Armachiho District	• 76% were fully immunized	High coverage
12	Tenaw G (2016)	Cross-sectional	288	Vaccination Coverage and Associated Factors	Debre Markos Town, Ethiopia	• 91.7% of children were completely vaccinated	High coverage
13	Yemesrach A(2016)	Cross-sectional	484	Predictors and Barriers to Full Vaccination among Children in Ethiopia	Worabe, SNNP, Ethiopia	• 61% were fully vaccinated	Relatively high coverage
14	Asrat M (2017)	Cross sectional	322	Assessment of Child Immunization Coverage and Associated Factors	Mizan Aman Town,	• 49.4% were partially immunized and 42.2% were fully immunized	Coverage was low
15	USAID(2015)	Cross-sectional	1597	Extended Program on Immunization (EPI) coverage in selected Ethiopian zones	Seven Zones, Ethiopia	• Penta III of 79% and fully 69% • Timely vaccination of 60%	Child vaccination coverage significantly varied among zones

**Fig. 2:**
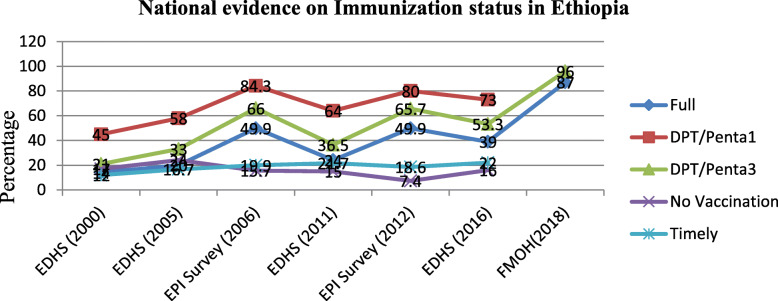
Trend of immunization coverage in Ethiopia

Timeliness of the valid doses given, as defined by timely doses administered before 12 months of age, was also assessed in the three studies conducted at national level. Evidenced from the national EPHI study indicated that, valid dose of all basic vaccines under one year was 18.6% by 2012 [5]. The EDHS 2016 report also has shown that only 22% of children were vaccinated timely before their first birth day [11]. These findings are lower than the full immunization coverage of similar studies indicating that children are not getting the recommended vaccines as per the WHO recommendations. The evidence also indicated that timeliness of immunization is not given due attention in the national EPI program [5, 11, 12]. The trend in immunization coverage also revealed that the immunization coverage is far below the target (Fig. 2). Timeliness was also assessed in one local study which indicated that the timely full immunization coverage was 60% that has much better performance as compared with the timeliness coverage reported by national studies [6]. Except the one mentioned, none of the local studies reported evidence on timeliness of immunization.

#### Determinants of immunization service utilization

Barriers and facilitators of immunization program were mainly tied to program acceptability, appropriateness, access and health system constraints. The main determinants associated with inequalities in coverage are multifaceted: From the existing evidence place of residence, region, maternal health services, access to media, distance from health facility and individual socio demographic characteristics of caregivers were found to be predictors of full immunization. There were a total of 6 national and 27 local studies on barriers and facilitators of immunization service uptake for which the findings are summarized below.

##### Geographic distribution

There were large geographical differences in vaccination coverage in Ethiopia as indicated by the national surveys [5, 10, 11, 32]. The immunization coverage in Afar, Somali and Gambella regions were much lower than the coverage’s in Addis Ababa and Diredawa [33, 34]. The consecutive EDHS surveys also indicated that regional disparities have not been changed over time [11]. The local studies also revealed that studies in Amhara and Oromia regions have better immunization coverage though they didn’t achieve the national targets set at national level [4]. The survey conducted by USAID in four regions of the country also indicated that there is significant variation in immunization coverage across regions and zones [6].

##### Household economic status

Household economic status strongly influences the likelihood that a child will be vaccinated. Children in the richest wealth quintile were more likely to be fully vaccinated when compared to children in the poorest quintile in majority of the reviewed studies [5, 6, 10, 11, 34]. In contrary, family income was found to be insignificant in a study from Arbegona district [35] and another study from SNNP region [28].

##### Age of caregivers/mothers

Majority of the studies showed that age of caregiver/mother has no significant association with immunization coverage [6, 19, 29, 36]. In contrary, from studies conducted in Jijiga town [26] and Arbegona district [35] it was found that it has a significant association with immunization coverage.

##### Birth order

The birth order of the child was not significant factor in two studies [31, 36] while it had significant association with child vaccination in the study from Arbegona district [35].

##### Family size

Family size was not addressed in most of the studies. Two studies concluded that family size has no significant association with immunization coverage [35, 36] while one study showed that it is a predictor for full immunization coverage [22].

##### Caregiver/mother’s education

Caregivers/mother’s educational status is an influential factor for using immunization services in all regions. Children of caregivers who have completed secondary or higher education are more likely to be vaccinated than children whose caregiver have no formal education [5, 6, 12, 20, 22, 25–29, 34].

##### Caregiver/mother’s occupation

Studies indicated that caregivers/mother’s occupation has no significant association with immunization service up take [12, 24, 29].

##### Child sex

In some societies with cultural discrimination against female children, boys have a greater chance to be vaccinated. In almost all studies child sex has no significant association with immunization coverage [6, 12, 19, 22, 28, 29, 31]. Only two studies concluded that it has significant association with child immunization [23, 24].

##### Place of residence

Place of residence measured as living in urban or rural area strongly influenced vaccination coverage in majority of the studies. Children in urban areas are significantly more likely to receive all recommended vaccinations than children in rural areas [5, 11, 12, 21, 23, 26–28]. On the other hand, effect of residence was not significant in three of the studies [6, 19, 29].

##### Knowledge about vaccination

Mothers knowledge was significantly associated with immunization coverage [19, 20, 22, 29, 36]. It was identified that children whose mothers had good knowledge on immunization and vaccine-preventable disease were more likely to be fully vaccinated than children whose mother has poor knowledge. This kind of knowledge can influence mothers’ health seeking behavior which in turn enhances immunization coverage. Knowledge on child vaccination was not significant predictor as indicated from the two studies [35, 36].

##### Attitude about vaccination

Positive attitude towards immunization was the enabling factors for full immunization [25]. Wrong perception on contraindication were significant predictors for partial immunization [18]. Similarly, wrong perception about vaccine side effects hinders immunization service uptake [35].

##### Access to media

Access to media and awareness about community conversation program were also predictors to full immunization coverage in two of the studies [28, 32] while it was found to be insignificant in one study [34].

##### Maternal health services

Attending ANC [19, 24, 32, 36, 37],TT vaccination [19, 23, 26], institutional delivery [19, 26, 29, 34, 36, 37] and PNC attendance [36] were found to be strong predictors of full immunization coverage. This could have happened due to mother’s health seeking behavior and it may create a good opportunity for the mother to vaccinate their children. On the other hand, in a study from Arbamich town and Zuria ANC follow up was not significant predictor of child immunization [29].

##### Geographic access

To increase coverage immunization service is supposed to be provided at static sites, outreach sites and through mobile approach for hard to reach areas. Short distance was enabler for full immunization [21, 24, 29] while distance to a functioning health facility did not show a difference in immunization coverage in another survey [5].

##### Household visit by health workers

House hold visit by health workers was not significant factor in one study [36] while it has a significant association with child immunization in another study [26].

##### Community level factors

Community level factors were not well addressed in majority of the studies. A study by Abadura et al. indicated that 21% of the variation in full immunization is attributed to community level factors. In this study, community ANC utilization rate has also significant association with full immunization coverage [34].

##### Reasons for vaccine hesitancy and not completing immunization

The reasons for not completing vaccination schedules were reported in some studies as descriptive findings. Among the reasons for defaulting, 41.8% was forgetting the appointment date and 34.2% lack of awareness [36]. The most common reasons for not vaccinating the child were fear of side effects (36%), being too busy (31%) and hearing rumors about vaccines (28%) [38]. Qualitative study in Hadiya Zone of Ethiopia also identified the main reasons for defaulting from the immunization program as poor counseling of mothers, unsupportive provider-client relationships and lack of systems for tracking defaulters [39].

### Health service availability

There were five studies which reported child immunization service availability. The evidence from EPHI indicated that 94% of public facilities offered child immunization services compared with 2% of private facilities [5]. In general, 80% of health facilities provide immunization service nationally. Regions wise, Benshangul-Gumuz, Tigray, Oromia, SNNP and Amhara regions have better coverage. On the other hand Addis Ababa has the lowest coverage [40] which could be explained by the fact that private health facilities are not engaged in routine immunization services. All the five studies revealed that most of health facilities are providing routine immunization service of which only few provide on daily basis [6, 40, 41].

According to EPHI survey in 2014, 53% of facilities that offer child immunization services have guidelines and 47% of them have at least one staff member trained on child immunization [41]. Actions by higher levels in conducting supervision and providing written feedback were the likely significant factors contributing to good immunization service performance in Ethiopia [42]. Facility level determinants including service interruption, training on EPI and defaulter tracing system were also independent predictors of complete vaccination [6] [Table 3].

**Table 3:** Evidence on child immunization service availability

S.N	Authors	Design	Sample size	Topic	Study area	Major findings /conclusions
1	EPHI (2012)	Cross sectional survey	585 government run health facilities	Ethiopian national immunization coverage survey	National	• 42.5% of health facilities had a planned session interrupted • Though more than 90% of the health facilities are providing routine EPI service, only 24.4% are providing the services daily • In-service training on EPI service delivery was low for health facility staff within the past year (57%) • The defaulter tracing system exists in 85% of health facilities
2	Habtamu B (2015)	Review	More than hundreds of related materials	Review on Measles Situation in Ethiopia; Past and Present	National	Accumulation of unvaccinated children in highly populated areas contributed for the frequent measles outbreaks occurring in different parts of the country
3	AschaleT(2014)	A cross-sectional study	302 health facilities	Factors contributing to routine immunization performance in Ethiopia	National	• Actions by higher levels in conducting supervision and providing written feedback are the likely significant factors contributing to good immunization performance in Ethiopia
4	EPHI (2014)	Cross sectional	835	Ethiopia Service Provision Assessment Plus Survey	National	• 53% of facilities that offer child immunization services have guidelines and 47% of them have at least one staff member trained • Majority of these facilities have equipment for vaccination services
5	USAID (2015)	Cross-sectional household and facility surveys	Selected health facilities	Extended Program on Immunization (EPI) coverage in selected Ethiopian zones	Seven Zones, Ethiopia	• 99% of health posts and 96% of health centers were providing RI • 37% of health centers were providing EPI services on a daily basis • Facility level determinants including service interruption, training on EPI and defaulter tracing system were independent predictors of complete vaccination
6	EPHI (2016)	Cross sectional	705 health facilities	SARA, Ethiopia	National	• 16% of facilities offered immunization services only in daily basis at the facility • Availability of the six antigens ranged between 29% for Oral Polio Vaccine to 36% for measles

### Supply chain management

The success of immunization program depends on reliable provision of commodities through the supply chain and availability for use when and where needed in the correct quantities and at the right time. The supply side determinants are key parts of immunization service provision and mainly controlled by the health care delivery system. The key indicators of supply side determinants include: availability of commodities and human resources. Commodity component is represented by the availability of functional refrigerators, cold box and vaccine in the health facilities. Availability of human resource for EPI is also examined as whether trained and dedicated staffs are available in the health facilities as per the national standard.

There have been six studies conducted on supply chain management. From EPHI survey 45.2% of health posts and 2.1% of health centers reported absence of vaccine refrigerator while 38.6% of health posts and 43.6% of health centers experienced stock-outs [5]. Another study also indicated that thermometer was not available in some of health centers (6%) and vaccine storage in the refrigerator was not proper in 73.4% centers [43]. Additionally, majority of the centers had neither trained personnel nor budget for maintenance of the cold chain [43]. Another survey from EPHI in 2016 reported that refrigerators and cold boxes were available in 31 and 71% of the health facilities [40]. Evidence from a study conducted in three regions showed that only 19% had functional refrigerators [44] and another study in Bale zone of Oromia region it was only 31% [45]. Vaccine storage in the refrigerator was not also proper in 54.5% facilities and 56% health workers had satisfactory knowledge on cold chain management [44]. Similarly 67% health centers and 40% health posts experienced shortage of vaccines [6] [Table 4].

**Table 4:** Evidences on supply chain management of immunization service in Ethiopia

S.N	Authors	Design	Sample	Topic	Study area	Major findings	Conclusions
1	Y. BERHANE (2000)	Institution based cross-sectional survey	67 health institutions providing static vaccination services	Cold chain status at immunization centers in Ethiopia	Addis Ababa city, and Hadiya Zones of southern Ethiopia	• Thermometer was not available in 6.3% • Vaccine storage in the refrigerator was not proper in 73.4% centers • Majority of the centers had neither trained personnel nor budget for maintenance of the cold chain	• Improving the maintenance conditions of refrigerators and • Introduction of cold chain monitoring devises are recommended
2	EPHI (2012)	Cross sectional survey	585 government-run health facilities	Ethiopian national immunization coverage survey	National	• 45.2% of health posts and 2.1% of health centers, reported absence of vaccine refrigerator • 38.6% of health posts and 43.6% of health centers experienced stock-outs	• Proper vaccine stock management is required
3	Roqie p (2012)	Institution based cross-sectional study	116 health facilities	Assessment of cold chain status for immunization in central Ethiopia.	Three districts (woredas) of Oromiya, SNNP and Amhara Regions	• Only 19% had functional refrigerators • Complete temperature recording of the last month was observed in 59.1% • Vaccine storage in the refrigerator was not proper in 54.5% facilities • 56% health workers had satisfactory knowledge on cold chain management	• There is an urgent need to improve knowledge and practice on cold chain management through improved supervision and training.
4	Bedasa Woldemichael 2013	Institution based cross-sectional study	183 health facilities	Cold Chain Status and Knowledge of Vaccine Providers at Bale Zone, Southeast Ethiopia:	Bale Zone, Southeast Ethiopia	• Only 31% health facilities had refrigerator • In 83% refrigerators thermometer was within the standard range	• There were gap in maintaining cold chain system and improper storage of vaccine were observed at study area
5	JSI L10k (2015)	Cross-sectional household and facility surveys	Selected health facilities	Extended Program on Immunization (EPI) coverage in selected Ethiopian zones: A baseline survey	Seven Zones, Ethiopia	• Almost all HCs and one-third of HPs had at least one refrigerator • Refrigerators were not functional in 32% health centers and 71% of HPs • 67% health centers and 40% health posts experienced shortage of vaccines	• In a significant proportion of facilities, cold chain management was suboptimal • Operational research to guide implementation
6	EPHI (2016)	Cross sectional	705 Health facilities	SARA, Ethiopia	National	• Refrigerators and cold boxes were available in 31 and 71% of HFs	• Low cold chain equipment’s

### EPI information systems

The quality of immunization and surveillance data should be regularly monitored and its use at each level should be promoted. Information generated from HMIS and surveys will be used for advocacy and for program and service improvement [4].

A total of six studies reported findings related with EPI information system. Two focused on surveillance while the remaining four on data management. The evidence showed that there was discrepancy between administrative reports and survey data indicating data quality problems. In addition, the evidence on surveillance and data management of immunization services were not adequate [46]. The reporting quality and information use of the EPI program for evidence based decision making deserve further concerted attention [47] [Table 12]. Vaccination cards are critical tools in ensuring that children receive all recommended vaccinations according to schedule. The 2016 EDHS found that only 46% of children age 12–23 months have vaccination cards [11] [Table 5].

**Table 5:** Evidences on EPI information system of Ethiopia

S.N	Authors	Design	Sample size	Topic	Study area	Major findings	Conclusions
1	Endriyas M(2014)	Retrospective cohort	2132 records	Poor quality data challenges conclusion and decision making	SNNP, Ethiopia	From a total of 2132 measles cases, 1319 (61.9%), had at least one dose of measles containing vaccine; the rest 398 (18.7%) and 415 (19.5%) were unvaccinated and had unknown status respectively	• Vaccination data or vaccine potency at lower level was unclear
2	JSI L10k (2015)	Cross-sectional surveys	Selected health facilities	Extended Program on Immunization (EPI) coverage	Seven Zones, Ethiopia	There was a 12% disparities in complete vaccination coverage between routine HMIS and survey coverage respectively	• Discrepancy in immunization data
3	Habtamu B(2015)	Review	More than hundreds of related materials	Review on Measles Situation in Ethiopia; Past and Present	National	Accumulation of unvaccinated children in highly populated areas contributed for the frequent measles outbreaks occurring in different parts of the country	• Working towards measleselimination and introduction of second dose measles vaccine in routine immunization program
4	Ketema Belda (2016)	Cross sectional	1059 suspected cases	Measles outbreak investigation in Guji zone of Oromia Region, Ethiopia	Guji zone, Oromia region	The cumulative attack rate of 81/100,000 population and case fatality ratio of 0.2% was recorded. Of these, 742 (70%) were zero doses of measles vaccine	• The case-based surveillance lacks sensitivity and timely confirmation of the outbreak
5	EPHI review (2016)	Cross sectional	544 Health facilities	Health Data Quality Review	National	From all facilities that report Penta3 immunization service data, 95% of facilities had completed data Overall, only 52% of the Penta3 data matched with the source documents	• Data quality problems observed
6	Liya W (2017)	Perspectives	Administrative data	Advances in the control of vaccine preventable diseases in Ethiopia	National	Surveillance data shows that cases of vaccine preventable diseases continue to occur in the country. During 2015 alone, more than 17,000 cases of measles were reported from throughout the country	• Ongoing efforts, adequate resources and capacity and new innovations and strategies continue to be needed

### Community engagement in immunization program

Community engagement is critical for demand generation and to improve quality of services. As part of the HEP packages, the community HDA has got due emphasis as it helps ensure greater involvement of individuals and communities in moving from supply-driven to demand-driven immunization services [3].

A total of eight studies were found in relation with community engagement. The evidence showed that the community engagement is generally poor though immunization service uptake is dependent on major factors: caretakers’ behavior, family characteristics and communication [48].

One study found that women’s awareness of community conversation program is the predictor of full immunization [37]. Another study also reported that 55, 53.8, and 84% of respondents had good knowledge, positive attitude, and good practice towards immunization of infants, respectively [49]. Similar study in Tigray region also indicated that households not visited by Health Extension Workers (HEWs) at least monthly; poor participation in women’s developmental groups and poor knowledge of child immunization were predictors of defaulting from vaccination [50]. A study conducted in Addis Ababa on vaccine hesitancy also showed that 3.4% reported ever hesitating and 3.7% ever refusing immunization service [38]. Provider-client relationship is also one of the factors affecting community engagement [39]. Existing health development army network and the regular meetings between the community and the health system actors were identified as a potential existing platform to harness community engagement [51] [Table 6].

**Table 6:** Evidence on community engagement for the immunization program

S.N	Author	Design	Sample	Topic	Study area	Major findings/Conclusions
1	Yihunie L (2011)	Cross-sectional	1927	Factors influencing full immunization coverage	National	• Women’s awareness of community conversation program is the predictor of full immunization
2	*Shiferaw B (2013)*	*Cross-sectional*	*634*	*Knowledge, Attitude and Practice of Mothers Towards* *Immunization of*	*Addis Ababa, Ethiopia*	• *Only 55.0, 53.8, and 84% of respondents had good knowledge, positive attitude, and good practice towards immunization of infants, respectively*
3	Hailay G (2015)	Case control study	90 cases and 180 controls	Determinants of defaulting from completion of child immunization	Laelay Adiabo District, Tigray	• Households not visited by HEWs; poor participation in women’s developmental groups and poor knowledge were predictors of defaulting
4	Chantler T (2016)	Formative evaluation with qualitative design	A total of 46 interviews and six FGDs	We All Work Together to Vaccinate the Child’: A Formative Evaluation of a Community-Engagement Strategy	Assosa and Bambasi woredas, Benshangual_Gumuz region	• The Enat Mastawesha calendar enabled health discussions between family member • Involving communities and relevant leaders in immunization programs can be very effective
5	Nina B (2017)	cross-sectional survey	350 caregivers	Vaccine hesitancy among caregivers and association with vaccination timeliness	Addis Ababa, Ethiopia	• 3.4% reported ever hesitating and 3.7% ever refusing Vaccine hesitancy increases the odds of untimely vaccination
6	*Asamne Z(2015)*	*A qualitative study*	*Twenty-six in-depth interviews*	*Reasons for defaulting from childhood* *immunization program: a qualitative study*	*Two districts of Hadiya zone, Southern Ethiopia*	• *The main reason for defaulting from the immunization was inadequate counseling of mothers and poor provider-client relationships*
7	Tefera T(2017)	A qualitative multiple case study design	63 focus group of 630 samples	Factors and misperceptions of routine childhood immunization service uptake in Ethiopia: findings from a nationwide qualitative study	National	• Lack of information at times of vaccination day and prolonged waiting time were the barriers • Significant misperceived benefits of immunization in the community • Immunization is dependent on major factors: caretakers’ behavior, family characteristics, information and communication
8	Binyam T (2017)	*Mixed methods approach*	*21 key informant interviews*	How can the use of data at each level of the health system be Increased to improve data quality, service delivery and shared accountability?	*North Gondar Zone, North West, Ethiopia*	• *Community engagement and shared accountability are important to improve immunization program*

### Gender inequalities to EPI services

Child gender preferences do not seem to play an important role for immunization inequalities in Ethiopia. Child sex was not significantly associated with child immunization in seven studies [6, 12, 19, 22, 28, 29, 31]. On the other hand, it was significant in two studies [23, 24].

At caregiver level, it was indicated that caregiver’s gender plays significant role as mothers are typically the primary caregivers of child immunization. The high workload on mothers compounded by the lack of support from male partners [39] and low decision power and autonomy in household level are the barriers for full immunization [25].

### Interventional studies on vaccination program

Implementation science is the study of methods to promote the adoption and integration of evidence-based practices, interventions and policies into routine health care and public health settings. Under this review, there were only four local level interventional studies. One study assessed the effectiveness of reminder sticker in reducing immunization dropout rate [52]. The other study was on Biomarker sero surveys that emphasized the importance of objective serological biomarker measurement in determining vaccination coverage surveys [53]. A technology based study that assessed the effect of text message reminders found no statistically significant association in improving immunization rates [54]. Another interventional study in Benshangul-Gumuz region indicated that Enat Mastawesha calendar as defaulter tracing mechanism was effective [55]. These findings point to the need for more implementation science research in the future to strengthen the immunization program in Ethiopia.

### Identified research priorities for the immunization program

The expert panel resulted in the specification of the main implementation challenges and identified the following priority areas for future research:

- Strengthening health facility-outreach service linkage
- Adoption of new technologies for the immunization program
- Availability of vaccines and supplies at health facility level
- Community based data verification mechanism for the immunization program
- Community engagement and professional-client communication
- Effectiveness of implementing eCHIS for immunization program
- Strategies to improve vaccine safety
- Women empowerment in immunization program
- Vaccination service provision in displaced community
- Revitalizing vaccination service in slum urban setting

## Conclusions

Regarding the current state of knowledge, the available evidence showed that there is adequate knowledge on full vaccination coverage and vaccination service availability. On the other hand, evidence on timeliness of vaccination, supply chain management, surveillance and data management of the immunization program are not adequate. There are minimal implementation science evidence nationally. Pertaining to barriers of the immunization program, there is adequate evidence on individual level factors while the evidence on health system and community level factors is scarce.

Although the proportion of fully vaccinated children is increasing over time, the existing evidence concluded that the national immunization coverage is below the target. Timeliness of immunization is found to be much lower than the full immunization coverage. The evidence from surveys, administrative reports and global estimates have also huge discrepancies. The evidence also indicated that there were large inequities in vaccination coverage related to socio-economic status, caregivers’ education, maternal health service uptake, access to media, knowledge about vaccination and distance to health facility. Child sex, age of the mother, family size and birth order had no consistent effect on child immunization across different settings.

Unlike in private ones, the routine child immunization service availability is better in public health facilities. The evidence on supply chain management also revealed that the cold chain is suboptimal and the availability of necessary equipment’s especially functional refrigerators is not as per the standard. There is also poor community engagement for the immunization program. In general, the factors that affect full immunization coverage vary from context to context which needs designing and implementing tailored interventions. Further research priorities are identified and there is a need to explore the remaining implementation barriers for the immunization program with more focus on the identified research priorities.

## Data Availability

The data sets extracted from the studies are available from the corresponding author upon reasonable request.

## Abbreviations

ANC: Ante Natal Care
CSA: Central Statistical Agency
cMYP: Comprehensive Multi Year Immunization Plan
EDHS: Ethiopian Demographic and Health Survey
EPHI: Ethiopian Public Health Institute
EPI: Expanded Program on Immunization
FMOH: Federal Ministry of Health
HMIS: Health Management Information System
HSTP: Health Sector Transformation Plan
PNC: Post Natal Care
RED: Reaching Every District
USIAD: United States Agency for International Development
VPD: Vaccine Preventable Disease
WHO: World Health organization
